# Photodynamic Therapy in Treatment of Oral Lichen Planus

**DOI:** 10.14740/jocmr2147w

**Published:** 2015-04-08

**Authors:** Diana Mostafa, Bassel Tarakji

**Affiliations:** a Department of Oral Maxillofacial Sciences, Al-Farabi College of Dentistry and Nursing, Riyadh, Kingdom of Saudi Arabia

**Keywords:** Oral lichen planus, Photodynamic therapy, Treatment

## Abstract

Oral lichen planus (OLP) is a relatively common chronic immunologic mucocutaneous disorder. Although there are many presenting treatments, some of them proved its failure. Recently, the use of photodynamic therapy (PDT) has been expanding due to its numerous advantages, as it is safe, convenient, and non-invasive and has toxic effect towards selective tissues. This article provides comprehensive review on OLP, its etiology, clinical features and recent non-pharmacological treatments. We also describe the topical PDT and its mechanisms. Our purpose was to evaluate the efficacy of PDT in treatment of OLP through collecting the data of the related clinical studies. We searched in PubMed website for the clinical studies that were reported from 2000 to 2014 using specific keywords: “photodynamic therapy” and “treatment of oral lichen planus”. Inclusion criteria were English publications only were concerned. In the selected studies of photodynamic treatment, adult patients (more than 20 years) were conducted and the OLP lesions were clinically and histologically confirmed. Exclusion criteria were classical and pharmacological treatments of OLP were excluded and also the using of PDT on skin lesions of lichen planus. We established five clinical studies in this review where all of them reported improvement and effectiveness of PDT in treatment of OLP lesions. The main outcome of comparing the related clinical studies is that the photodynamic is considered as a safe, effective and promising treatment modality for OLP.

## Introduction of Oral Lichen Planus (OLP)

OLP is a relatively common chronic, immunologic, inflammatory, mucocutaneous disorder. It affects 0.5-2% of general population. It has higher incidence in females with no racial predisposition. It affects middle-aged patients (over 35 years) while children and teenagers are rarely affected. Oral form of lichen planus occurs more frequently and tends to be more resistant to treatment than cutaneous form. Approximately one-third of the patients who have oral lesions also have skin lesions [1-8].

### Etiology

There are several hypotheses involving genetic, infectious, psychogenic and autoimmune factors [9]. Recently, it was accepted that OLP involves chronic cell-mediated immune reaction of unknown etiology that induces damage to basal cell layer of the epithelium, in which T lymphocytes accumulate in the superficial lamina propria, basement membrane (BM) disruption, intra-epithelial T-cell migration and keratinocyte apoptosis in OLP [10, 11]. It was reported that patients with OLP have higher levels of stress, anxiety, and depression. Elevations of salivary cortisol (stress hormone) and psychological factors have been found in OLP patients. It was also found that local conditions such as poor oral hygiene and smoking may exacerbate OLP [9, 12-15]. Also, there are many reports of association between erosive OLP and infectious agents: herpes simplex virus (HSV), Epstein-Barr virus (EBV), human papilloma virus (HPV), cytomegalovirus (CMV) and human immunodeficiency virus (HIV). It could be due to the persistence of infection in the epithelial cells affected with viruses [13, 16, 17].

A number of previous reports have suggested that patients with OLP have associated diabetes, hypertension and liver diseases more often than patients without OLP [18, 19]. Conversely, some reports have not supported these presumptions [20]. Also, more than 90 controlled studies had reported association between OLP and hepatitis C virus [13, 16, 21]. The probable reason is the ability of HCV-RNA for cytopathic replication outside the liver cells, persistent infection and triggering of immunological processes (the activated CD8 T cells, cytokines and expansion of certain B-cell clones), leading to OLP manifestations [22]. The CD8^+^ T cell proportion of OLP-HCV patients in lamina propria was significantly higher and deeper than in OLP which gives the more aggressive erosive manifestation of OLP-HCV patients [23]. On the other hand, this association was not confirmed by Dupin et al [24], who stated that there was no association between OLP and HIV.

### Clinical features

OLP has characteristic clinical features and distribution in the oral mucosa. It is commonly presented bilaterally on posterior buccal mucosa but not always symmetrical. OLP lesions are characterized by periods of exacerbation and remission [25, 26]. The OLP lesion is based on six clinical forms that were described by Andreason [27] as reticular, papular, plaque, atrophic, erosive, and bullous. The same patient may have OLP with multiple forms [28]. Reticular OLP is the most common form that is characterized by asymptomatic interlacing white keratotic lines called Wickham’s striae where it does not disappear by stretching or rubbing its surface [29]. Erosive OLP is a mix of erythematous and ulcerated areas. Atrophic OLP is diffuse erythematous patches of mucosal thinning and combined with striae. Bullous OLP has intraoral bullae that rupture soon after their appearance, causing painful ulcerated surface. Plaque OLP is described as homogenous white patches looking like oral keratosis. Papular OLP contains small white papules with fine striae [27]. The cutaneous lesions can be described as shiny, well-defined, purplish, polygonal, planar, pruritic papules and plaques that may reveal white striations (Wickham’s striae). The flexor surfaces of the legs and arms, especially the wrists are the most common sites [30, 31]. Malignant potential of OLP is estimated 0.4-6.25%. Only lesions that have dysplasia are potentially at risk of developing into cancer. Smoking and alcohol may increase the risk of oral cancer [32-34]. The incidence of squamous cell carcinoma is higher for females. The malignant transformation occurs most frequently in the erosive form of OLP 35%, followed by the plaque type 24% and the reticular type 11% [35-37].

### Recent non-pharmacological treatment of OLP

Due to the partially understood pathogenic mechanism and potential malignant nature of OLP [32], various modalities are done for treatment of the symptomatic OLP. The aim of these modalities is to reduce the length and severity of symptomatic outbreaks [30]. Follow-ups and periodic examinations are needed to avoid any possible malignant transformation [35, 38].

Lasers have been suggested as non-pharmacological modality to patients resistant to conventional treatment but their effectiveness is under study [39]. Lasers are used in high- or low-level laser irradiation manners for treatment of OLP. Some studies have conducted that high-level lasers are used for successful OLP tissue ablation such as CO_2_ lasers which have been proven to be very effective treatment for OLP with decreasing recurrence rate 20% in which the thermal laser energy carbonizes superficial layers of epithelium [40, 41].

However, low dose 308-nm excimer laser is a palliative treatment when the mucosa lacks the overlying epithelial layer, and a higher and more effective UV dose is allowed to reach the infiltrating lymphocytes [42]. Diode laser 980 nm was also reported as an easy, effective, fast and safe treatment of OLP [39].

Photodynamic therapy (PDT) has been reported as a very promising treatment method as it does not cause scarring because of the eradication of microfoci after PDT, which cannot be seen upon macroscopical examination. It also decreases risk of recurrence [43, 44].

## The PDT

PDT is a recent therapy that began to be explored in treatment of OLP. It is a special type of treatment that combines two components: a photosensitive agent or photosensitizer (PS) and a harmless light source of a specific wavelength [45]. The visible light irradiation forms biochemical interactions in the presence of the PS which undergoes excitation and produces toxic oxygen species such as singlet oxygen and free radical that cause specific cellular destruction, membrane lysis and protein inactivation [46-48]. Nowadays, PDT is used for the treatment of various types of oral diseases such as cancers, leukoplakia, erythroleukoplakia, dysplasia, and mucosal hypertrophy. Its efficacy varies, from complete regression of lesions to the lack of response to treatment [49].

### PS used in PDT

PDT PSs are chemical compounds that can be promoted to an excited state upon absorption of the light. When they capture light energy, they transfer it into chemical reaction in the presence of molecular oxygen produces singlet oxygen (^1^O_2_) or superoxide (O_2_^-^), which gives them the ability to induce cell damage through direct and indirect cytotoxicity [50, 51].

PSs can be classified by their chemical structures and origins into three broad families: 1) porphyrin-based PS (e.g. photofrin, 5-aminolevulinic acid (ALA/PpIX), BPD-MA), 2) chlorophyll-based PS (e.g. chlorins, purpurins, bacteriochlorins), and 3) dye (e.g. phtalocyanine, napthalocyanine). Most of the currently approved clinical PSs belong to the porphyrin family [51].

The first PSs used in PDT were compounds belonging to the group of hematoporphyrin, photofrin, and meta-tetra (hydroxyphenyl) chlorin. After extended period of time, the PSs of choice included ALA and dyes - toluidine blue and methylene blue (MB) [52].

Methyl 5-aminolevulinate (MAL) is an esterified derivative of ALA. It is lipophilic and its selectivity for specific cells is greater than that of ALA which increases its phototoxicity effect [53].

The phenothiazine dye MB has excellent photochemical properties and is recognized as non-toxic dye. Unlike other PSs, it can be administrated topically and orally, which make it the preferred choice for superficial lesions in skin and oral cavity [54-56]. It also has high absorption of wavelengths at 665 nm, where light penetration into the tissue is optimal [54, 57, 58]. It has a natural antifungal and antibacterial activity, of which toxic potential can be increased by light activation [54, 55, 59].

Photolon^®^, a novel chlorine 6-derived PS, is a pigment that has been successfully substantiated with evidence in the treatment of precancerous, cancerous lesions [49, 60, 61], and it had produced advantageous outcomes in OLP treatment, reducing the main signs and symptoms even without the need for analgesics or anesthetics during PDT which make it suitable choice of treatment for the old patients [49].

However, until this moment, there is no PS that would have all clinical demands. Most of them have disadvantages like: prolonged skin photosensitivity, limited ability of cell selectivity, and unpredictable efficacy [57].

### Types of lights source used in topical PDT

Various light sources are used in topical PDT. 1) Lasers are considered as an ideal light source for PDT due to its coherence and monochromaticity. The therapy of soft tissue performed with such lasers is called “low level laser therapy” (LLLT) [62]. Nowadays, diode lasers are commonly used in clinics. They are compact and portable. They have a high conversion efficiency from electrical energy to laser energy [62-64]. 2) Lamps have been useful in PDT especially in the cure of skin diseases such as metal halogen lamp and short arc xenon lamp. Although the light is filtered, the wavelength bands are wide compared with the absorption bands of the photosensitizing substances which lead to heat release that causes the cytotoxic effect [63]. 3) Lasers emitted diodes (LEDs) generate wavelength bands wider than those from lasers [63].

### Mechanism of PDT

This treatment is done in two stages. In the first stage, the photosensitizing agent is accumulated in the target cells (rapidly dividing cells) that need to be treated, following topical administration. This was explained by disproportionately of high numbers of low density lipoproteins receptors of their cell membranes and abnormal microvasculature [65]. However, not only this type of tissues exhibit accumulation of PS but also there are microorganisms like bacteria, fungi and viruses exhibiting selective accumulation of PSs due to differences in permeability of their outer structures. In addition, several normal body tissues exhibit the same phenomenon which is not well understood [65, 66].

While in the second stage, the photosensitized cells are exposed to light source of specific wavelength that coincides with the absorption spectrum of the PS. The second step is: a measured light dose of appropriate wavelength is then used to irradiate the target tissue [67, 68]. This light activates the dye and elicits a series of cytotoxic reactions. It causes the generation of reactive oxygen species (ROS) that has toxic effect on target cells [69-71].

PDT produces cytotoxic effects by three mechanisms: cellular, vascular and immunological responses. Combination of these responses depends on the tissue oxygen availability, the PS and the illumination scheme used [72, 73].

When PS localizes in cell membranes, it causes interruption and damage to plasma membrane that may release harmful proteins and chemicals. Then, it causes damage to adjacent cells [74]. PSs which are localized in mitochondria produce cell death via apoptosis, while those localized in lysosomes and plasma membranes induce cell death predominantly by necrosis [75]. They do not accumulate in the nucleus which makes them have low potential to generate DNA mutations or carcinogenic effect [75]. Changes in vessel diameter, platelet aggregation and leakage of vessels during PDT cause the formation of edema [76, 77]. This causes indirect damage by the deprivation of oxygen and nutrients which affects the effectiveness of PDT. Without the availability of oxygen and singlet oxygen, PDT cannot be induced [72, 76, 77]. Also PDT affects the immune system and cytokines, chemokines and other biological response modifiers are released. The inflammatory cytokines interleukin IL-6 and IL-10 but not TNF-α have been discovered to be regulated after PDT. Also, neutrophils were increased in number after PDT [76, 78-80].

## Discussion

The treatment of symptomatic OLP represents a therapeutic challenge. Despite numerous existing remedies, some of these treatments are failures. Recently, the use of PDT has been expanding due to its numerous advantages, as it is safe, convenient and non-invasive as it has selective toxicity towards target tissues. It has also excellent cosmetic results, where healing produced with little or no scarring. It can be repeated without producing any harm to normal tissues and can be used alone or in conjunction with other treatment [81].

Aghahosseini et al [52] in 2006 estimated that PDT is an alternative method for the treatment of OLP in 13 patients with 26 mucosa lesions. The patients rinsed with 5% aqueous solution of MB dye for 5 min. After 10 min, the lesions were exposed to a low-energy laser of 632 nm wavelength and exposure dose of 120 J/cm^2^ (Table 1). Sixteen lesions were improved and four lesions had complete remission. The mean reduction in the size of lesions was 44.3%. The same author published in another research of case report, the application of PDT mediated by MB in two patients had five lichen planus lesions (Table 1). Two lesions completely disappeared, two lesions were improved 50% clinically and one lesion had no response [82].

**Table 1:** The Published Clinical Researches of Photodynamic Therapy in Treatment of Oral Lichen Planus From 2000 to 2014

Author	Type of the study	Number of patients	Light source of PDT	Photosensitizer used in PDT
Sadaksharam et al, 2012 [48]	Case report	20	Xenon arc lamp of 630 ± 5 nm and 120 J/cm^2^)	Methylene blue
Sobaniec et al, 2013 [49]	Case report	23	Diode laser (660 nm and 90 J/cm^2^)	Chlorine-e-6 polyvinyl pyrrolidone (Photolon^®^)
Aghahosseini et al, 2006 [52]	Case report	13	Diode laser (632 nm and 120 J/cm^2^)	Methylene blue
Aghahosseini et al, 2006 [82]	Case report	2	Diode laser (632 nm and 100 J/cm^2^)	Methylene blue
Kvaal et al, 2013 [83]	Case report	14	Diode laser (600 - 660 nm and 75 J/cm^2^)	Methyl 5-aminolevulinate

Sadaksharam et al [48] in 2012 (Table 1) conducted a research on 20 patients with systemic OLP. The patients were treated by PDT using xenon arc lamp of 630 ± 5 nm wavelength and total dose of 120 J/cm^2^ per sitting in four sessions mediated by MB. They achieved a significant reduction in lesions over prolonged period without any side effects. This was also supported by Sobaniec et al [49] who concluded that there were no adverse effects during PDT or during the follow-up periods.

Sobaniec et al [49] (Table 1) obtained a clinical study on 23 patients with 48 lesions treated with PDT using gel containing 20% chlorine-e-6 Photolon^®^ and 10% dimethyl sulfoxide which was applied directly onto the lesion and the surrounding healthy mucosa 1 h before exposure to a semiconductor laser with wavelength 660 nm. A series of illuminations were performed using light energy density of 90 J/cm^2^. The appointments were scheduled at 2-week intervals, but no longer than for 10 sessions. They concluded that PDT could be useful in treatment of OLP where the size of clinical lesions in patients decreased significantly in 55%, and the best effects were observed on lining mucosa more than masticatory mucosa.

Sobaniec et al [49] and Aghahosseini et al [52] (Table 1) had used the diode laser with the same wavelength and energy density but different PSs but Aghahosseini et al [52] estimated that the significant improvement of signs and symptoms was after 1 week of a single session of PDT and during the follow-up period (up to 12 weeks). This was confirmed by Kvaal et al [83] who conducted a clinical study on 14 patients treated by PDT mediated by a porphyrin, MAL (Table 1). The OLP lesions were illuminated by diode laser with wavelength of 600 - 660 nm and energy density of 75 J/cm^2^ to treat OLP patients for 6 months. They reported remarkable lasting improvement after a single treatment, and even during 4 years of follow-up, without any side effects [83, 84].

## Conclusion

In conclusion, the main outcome of comparing the clinical studies is that the PDT is considered as a safe and effective advanced treatment modality for OLP. PDT is giving a remarkable effectiveness and improvement evidences in treatment of OLP where the selected articles demonstrated a significant reduction in the signs and symptoms of OLP and an increase in the symptom-free periods.

Due to the confined number of relevant published data of PDT, limited sample size and short follow-up periods, we could not give definite evidence for the advantage of PDT in treatment of OLP. More clinical studies and verifications with randomized clinical trials, larger numbers of patients and long follow-up periods are needed to evaluate the effectiveness of PDT in treatment of OLP.

## References

[1] Bobbio A, Vescovi P, Ampollini L, Rusca M (2007). Oral erosive lichen planus regression after thymoma resection. Ann Thorac Surg.

[2] Mollaoglu N (2000). Oral lichen planus: a review. Br J Oral Maxillofac Surg.

[3] Cawson R, Odell W (2008). Diseases of oral mucosa: Non-infective stomatitis. Cawson's Essentials of Oral Pathology and Oral Medicine.

[4] Alam F, Hamburger J (2001). Oral mucosal lichen planus in children. Int J Paediatr Dent.

[5] Greenberg M, Glick M (2008). Red and white lesions of oral mucosa. Burket's Oral Medicine Diagnosis and Treatment.

[6] Thongprasom K, Youngnak-Piboonratanakit P, Pongsiriwet S, Laothumthut T, Kanjanabud P, Rutchakitprakarn L (2010). A multicenter study of oral lichen planus in Thai patients. J Investig Clin Dent.

[7] Scully C, Carrozzo M (2008). Oral mucosal disease: Lichen planus. Br J Oral Maxillofac Surg.

[8] Scattarella A, Petruzzi M, Ballini A, Grassi F, Nardi G (2011). Oral lichen planus and dental hygiene: a case report. Int J Dent Hyg.

[9] Sheikh MA, Nazir AK, Rasheed A (2003). Oral lichen planus. JK Science.

[10] Thornhill MH (2001). Immune mechanisms in oral lichen planus. Acta Odontol Scand.

[11] Sugerman PB, Savage NW, Walsh LJ, Zhao ZZ, Zhou XJ, Khan A, Seymour GJ (2002). The pathogenesis of oral lichen planus. Crit Rev Oral Biol Med.

[12] Shekar C, Ganesan S (2011). Oral Lichen Planus: review. Journal of Dental Sciences & Research.

[13] Mahboobi N, Agha-Hosseini F, Lankarani KB (2010). Hepatitis C virus and lichen planus: the real association. Hepat Mon.

[14] Akay A, Pekcanlar A, Bozdag KE, Altintas L, Karaman A (2002). Assessment of depression in subjects with psoriasis vulgaris and lichen planus. J Eur Acad Dermatol Venereol.

[15] Shah B, Ashok L, Sujatha GP (2009). Evaluation of salivary cortisol and psychological factors in patients with oral lichen planus. Indian J Dent Res.

[16] Carrozzo M, Thorpe R (2009). Oral lichen planus: a review. Minerva Stomatol.

[17] Scully C, Eisen D, Carrozzo M (2000). Management of oral lichen planus. Am J Clin Dermatol.

[18] Ali AA, Suresh CS (2007). Oral lichen planus in relation to transaminase levels and hepatitis C virus. J Oral Pathol Med.

[19] Canjuga I, Mravak-Stipetic M, Loncar B, Kern J (2010). The prevalence of systemic diseases and medications in patients with oral lichen planus. Acta Stomatol Croat.

[20] Machado AC, Sugaya NN, Migliari DA, Matthews RW (2004). Oral lichen planus. Clinical aspects and management in fifty-two Brazilian patients. West Indian Med J.

[21] Chung CH, Yang YH, Chang TT, Shieh DB, Liu SY, Shieh TY (2004). Relationship of oral lichen planus to hepatitis C virus in southern Taiwan. Kaohsiung J Med Sci.

[22] Ghabanchi J, Maleki SR, Afkar MD (2009). Prevalence of Oral lichen planus in HCV+ Patients in Shiraz, South of Iran Middle East. Journal of Digestive Diseases.

[23] Nagao Y, Sata M, Noguchi S, Seno'o T, Kinoshita M, Kameyama T, Ueno T (2000). Detection of hepatitis C virus RNA in oral lichen planus and oral cancer tissues. J Oral Pathol Med.

[24] Dupin N, Chosidow O, Lunel F, Fretz C, Szpirglas H, Frances C (1997). Oral lichen planus and hepatitis C virus infection: a fortuitous association?. Arch Dermatol.

[25] Eisen D, Carrozzo M, Bagan Sebastian JV, Thongprasom K (2005). Number V Oral lichen planus: clinical features and management. Oral Dis.

[26] Ismail SB, Kumar SK, Zain RB (2007). Oral lichen planus and lichenoid reactions: etiopathogenesis, diagnosis, management and malignant transformation. J Oral Sci.

[27] Andreasen JO (1968). Oral lichen planus. 1. A clinical evaluation of 115 cases. Oral Surg Oral Med Oral Pathol.

[28] Canto AM, Muller H, Freitas RR, Santos PS (2010). Oral lichen planus (OLP): clinical and complementary diagnosis. An Bras Dermatol.

[29] Martin S, Michael G (2003). Burket’s Oral Medicine.

[30] Edwards PC, Kelsch R (2002). Oral lichen planus: clinical presentation and management. J Can Dent Assoc.

[31] Wolff K, Johnson RA, Suurmond D (2005). Fitzpatrick’s Color Atlas & Synopsis of Clinical Dermatology.

[32] Hosni ES, Yurgel LS, Silva VD (2010). DNA ploidy in oral lichen planus, determined by image cytometry. J Oral Pathol Med.

[33] Bromwich M (2002). Retrospective study of the progression of oral premalignant lesions to squamous cell carcinoma: a South Wales experience. J Otolaryngol.

[34] Neville BW, Damm DD, Allen CM, Bouquot JE (2009). Oral & Maxillofacial Pathology.

[35] Irani S (2010). Squamous cell carcinoma arising in oral lichen planus. DJH.

[36] Mattsson U, Jontell M, Holmstrup P (2002). Oral lichen planus and malignant transformation: is a recall of patients justified?. Crit Rev Oral Biol Med.

[37] Neppelberg E Pathological Mechanisms in Oral Lichen Planus. PhD thesis. Oral Sciences, Oral Pathology and Oral Medicine, Faculty of Dentistry. Norway: University of Bergen, 2007.

[38] Chainani-Wu N, Silverman S, Jr., Lozada-Nur F, Mayer P, Watson JJ (2001). Oral lichen planus: patient profile, disease progression and treatment responses. J Am Dent Assoc.

[39] Desiate A, Cantore S, Tullo D, Profeta G, Grassi FR, Ballini A (2009). 980 nm diode lasers in oral and facial practice: current state of the science and art. Int J Med Sci.

[40] Deeppe H, Hillemanns M, Hauck W (2004). Recurrence rates of premalignant lesions after CO2 laser vaporization. Med Laser.

[41] Deppe H, Horch HH, Henke J, Donath K (2001). Peri-implant care of ailing implants with the carbon dioxide laser. Int J Oral Maxillofac Implants.

[42] Trehan M, Taylor CR (2004). Low-dose excimer 308-nm laser for the treatment of oral lichen planus. Arch Dermatol.

[43] Sieron A, Adamek M, Kawczyk-Krupka A, Mazur S, Ilewicz L (2003). Photodynamic therapy (PDT) using topically applied delta-aminolevulinic acid (ALA) for the treatment of oral leukoplakia. J Oral Pathol Med.

[44] Jurczyszyn K, Zikowski P, Gerber H, Osiecka BJ (2007). Potentiality of photodynamic therapy in dentistry-literature review. Dent Med Probl.

[45] Nelke KH, Pawlak W, Leszczyszyn J, Gerber H (2014). Photodynamic therapy in head and neck cancer. Postepy Hig Med Dosw.

[46] Jori G, Fabris C, Soncin M, Ferro S, Coppellotti O, Dei D, Fantetti L (2006). Photodynamic therapy in the treatment of microbial infections: basic principles and perspective applications. Lasers Surg Med.

[47] Meisel P, Kocher T (2005). Photodynamic therapy for periodontal diseases: state of the art. J Photochem Photobiol B.

[48] Sadaksharam J, Nayaki KP, Selvam NP (2012). Treatment of oral lichen planus with methylene blue mediated photodynamic therapy--a clinical study. Photodermatol Photoimmunol Photomed.

[49] Sobaniec S, Bernaczyk P, Pietruski J, Cholewa M, Skurska A, Dolinska E, Duraj E (2013). Clinical assessment of the efficacy of photodynamic therapy in the treatment of oral lichen planus. Lasers Med Sci.

[50] Skovsen E, Snyder JW, Lambert JD, Ogilby PR (2005). Lifetime and diffusion of singlet oxygen in a cell. J Phys Chem B.

[51] Huang Z (2005). A review of progress in clinical photodynamic therapy. Technol Cancer Res Treat.

[52] Aghahosseini F, Arbabi-Kalati F, Fashtami LA, Djavid GE, Fateh M, Beitollahi JM (2006). Methylene blue-mediated photodynamic therapy: a possible alternative treatment for oral lichen planus. Lasers Surg Med.

[53] Morton CA, Brown SB, Collins S, Ibbotson S, Jenkinson H, Kurwa H, Langmack K (2002). Guidelines for topical photodynamic therapy: report of a workshop of the British Photodermatology Group. Br J Dermatol.

[54] Chen Y, Zheng W, Li Y, Zhong J, Ji J, Shen P (2008). Apoptosis induced by methylene-blue-mediated photodynamic therapy in melanomas and the involvement of mitochondrial dysfunction revealed by proteomics. Cancer Sci.

[55] Tardivo JP, Del Giglio A, de Oliveira CS, Gabrielli DS, Junqueira HC, Tada DB, Severino D (2005). Methylene blue in photodynamic therapy: From basic mechanisms to clinical applications. Photodiagnosis Photodyn Ther.

[56] Allison RR, Downie GH, Cuenca R, Hu XH, Childs CJ, Sibata CH (2004). Photosensitizers in clinical PDT. Photodiagnosis Photodyn Ther.

[57] De Paula LF, Santos RO, Menezes HD, Vieira Jr JB (2010). A Comparative Study of Irradiation Systems for Photoinactivation of Microorganisms. J Braz Chem Soc.

[58] Junqueira HC, Severino D, Dias LG, Gugliotti M, Baptista MS (2002). Modulation of the methylene blue photochemicalproperties based on the adsorption at aqueous micelle interfaces. Phys Chem Chem Phys.

[59] Wainwright M, Crossley KB (2002). Methylene Blue--a therapeutic dye for all seasons?. J Chemother.

[60] Juzeniene A (2009). Chlorin e6-based photosensitizers for photodynamic therapy and photodiagnosis. Photodiagnosis Photodyn Ther.

[61] Trukhachova TV, Shliakhtsin SV, Isakov GA, Istomin YP (2008). Photolon® (Fotolon®) - a novel photosensitizer for photodynamic therapy. A review of physical, chemical, pharmacological and clinical data. Minsk.

[62] Kulekcioglu S, Sivrioglu K, Ozcan O, Parlak M (2003). Effectiveness of low-level laser therapy in temporomandibular disorder. Scand J Rheumatol.

[63] Calin MA, Parasca SV (2006). Photodynamic therapy in oncology. Journal of Optoelectronics and Advanced Materials.

[64] Walsh LJ (2003). The current status of laser applications in dentistry. Aust Dent J.

[65] Costa RA, Farah ME, Cardillo JA, Belfort R, Jr (2001). Photodynamic therapy with indocyanine green for occult subfoveal choroidal neovascularization caused by age-related macular degeneration. Curr Eye Res.

[66] Koh AH, Ang CL (2002). Age-related macular degeneration: what's new. Ann Acad Med Singapore.

[67] Konan YN, Gurny R, Allemann E (2002). State of the art in the delivery of photosensitizers for photodynamic therapy. J Photochem Photobiol B.

[68] Dougherty TJ, Gomer CJ, Henderson BW, Jori G, Kessel D, Korbelik M, Moan J (1998). Photodynamic therapy. J Natl Cancer Inst.

[69] Donnelly RF, McCarron PA, Woolfson D (2009). Drug delivery systems for photodynamic therapy. Recent Pat Drug Deliv Formul.

[70] Ochsner M (1997). Photophysical and photobiological processes in the photodynamic therapy of tumours. J Photochem Photobiol B.

[71] Moan J, Streckyte G, Bagdonas S, Bech O, Berg K (1997). Photobleaching of protoporphyrin IX in cells incubated with 5-aminolevulinic acid. Int J Cancer.

[72] Kessel D, Reiners JJ, Jr (2007). Apoptosis and autophagy after mitochondrial or endoplasmic reticulum photodamage. Photochem Photobiol.

[73] Manda G, Nechifor MT, Neagu TM (2009). Reactive oxygen species, cancer and anti-cancer therapies. Curr Chem Biol.

[74] Hasan T, Ortel B, Frei H (2003). Photodynamic therapy of cancer. Chapter 40. Cancer Medicine.

[75] Korbelik M (2006). PDT-associated host response and its role in the therapy outcome. Lasers Surg Med.

[76] Oleinick NL, Morris RL, Belichenko I (2002). The role of apoptosis in response to photodynamic therapy: what, where, why, and how. Photochem Photobiol Sci.

[77] Xia C, Wang Y, Chen W, Yu W, Wang B, Li T (2011). New hydrophilic/lipophilic tetra-alpha-(4-carboxyphenoxy) phthalocyanine zinc-mediated photodynamic therapy inhibits the proliferation of human hepatocellular carcinoma Bel-7402 cells by triggering apoptosis and arresting cell cycle. Molecules.

[78] Hopper C (2000). Photodynamic therapy: a clinical reality in the treatment of cancer. Lancet Oncol.

[79] Jerjes W, Upile T, Betz CS, El Maaytah M, Abbas S, Wright A, Hopper C (2007). The application of photodynamic therapy in the head and neck. Dent Update.

[80] Rad M, Hashemipoor MA, Mojtahedi A, Zarei MR, Chamani G, Kakoei S, Izadi N (2009). Correlation between clinical and histopathologic diagnoses of oral lichen planus based on modified WHO diagnostic criteria. Oral Surg Oral Med Oral Pathol Oral Radiol Endod.

[81] Gale N, Pilch BZ, Sidransky D, Eveson J (2005). WHO classification of tumors: pathology and genetics of tumors of the head and neck.

[82] Aghahosseini F, Arbabi-Kalati F, Fashtami LA, Fateh M, Djavid GE (2006). Treatment of oral lichen planus with photodynamic therapy mediated methylene blue: a case report. Med Oral Patol Oral Cir Bucal.

[83] Kvaal SI, Angell-Petersen E, Warloe T (2013). Photodynamic treatment of oral lichen planus. Oral Surg Oral Med Oral Pathol Oral Radiol.

[84] Kvaal SI, Warloe T (2007). Photodynamic treatment of oral lesions. J Environ Pathol Toxicol Oncol.

